# Observations on the Term of Utero-gestation in Man and Inferior Animals

**Published:** 1847-10

**Authors:** T. T. Lockwood


*Observations on the term of Utero-gestation in man and inferior animals, &c.* By T. T. Lockwood, M. D.—The following statistics, which have been collected with accuracy, may possess interest for some readers. My attention having been directed to the subject by a little incident occurring in my neighborhood, I was led to consult authorities on the term of utero-gestation, and I found not a little discrepancy of opinion. I then commenced keeping a note book of the term in some of the domestic animals, and the following results, relating to the Cow, are submitted:

In six hundred and twenty-one Cows, fifty calved between two hundred and sixty and two hundred and seventy days; five hundred and fifty-six, between two hundred and seventy and two hundred and eighty days; fourteen between two hundred and eighty and two hundred and eighty-six; one, only, went two hundred and ninety days.

Five hundred and fifty of the cows were observed to have the mucous discharge, more or less, for twenty-four hours previous to calving. All that were noticed seemed to be very restless for twelve or fourteen hours previous, and when labor came on seemed to have regular pains.

Most of those that calved short of two hundred and seventy days, were heifers with their first calves; and all of them that went over two hundred and eighty days, were old cows with large abdomens.

The conclusion from these observations is, that a cow seven years old, and well built, will most probably go two hundred and seventy-six days.

The following experiments were made to ascertain the importance of the condition of *heat* in the same animal:—A two year old heifer was subjected to involuntary intercourse twice, and kept separate for the rest of the year. Conception did not follow. This experiment was tried in three instances with the same result.

The actual duration of the term of gestation in the human subject was ascertained in the following cases:—

——, aged 19, duration 272 days, first confinement. ——, aged 30, first confinement, duration 276 days. ——, aged 17, duration 270 days. ——, aged 44, seventh confinement, duration 284 days, the child weighing fourteen pounds.—*Buffalo Med. Journal.*

